# Study of the expression of CD30 in pterygia compared to healthy conjunctivas

**Published:** 2009-10-17

**Authors:** Yonathan Garfias, Víctor Manuel Bautista-De Lucio, Cynthia García, Angel Nava, Leonardo Villalvazo, María Carmen Jiménez-Martínez

**Affiliations:** 1 Research Unit, Institute of Ophthalmology “Conde de Valenciana” Mexico City, Mexico; 2 Oculoplastics Department, Institute of Ophthalmology “Conde de Valenciana” Mexico City, Mexico; 3 Pathology Department, Institute of Ophthalmology “Conde de Valenciana” Mexico City, Mexico; 4 Department of Immunology, Institute of Ophthalmology “Conde de Valenciana” Mexico City, Mexico; 5 Department of Biochemistry, Faculty of Medicine, National Autonomous University of Mexico, Mexico City, Mexico

## Abstract

**Purpose:**

The pterygium is characterized by a fibrovascular neoformation from the bulbar conjunctiva into the cornea. The recent discovery that abnormal markers associated with tumor diseases are identified in the pterygium strengthens the theory that the pterygium is a tumor-like disease rather than a degenerative disease. The CD30 molecule has been identified in neoplastic and normal epithelial proliferating cells. The aim of this study was to determine the expression of the CD30 molecule in the pterygium.

**Methods:**

Immunohistochemical staining using an antibody to CD30 and to the Ki-67 nuclear antigen was performed on 25 pterygial specimens and 10 healthy conjunctivas.

**Results:**

Strong immunostaining against CD30 was observed in all pterygium specimens, in contrast to the healthy conjunctivas that showed weak immono-positivity in only three cases. The staining was diffused, predominantly to the basal epithelium. The Ki-67 antigen was observed in the nucleus of the basal epithelium of the pterygial specimens, and no staining was observed in the healthy conjunctiva. When serial sections were stained with CD30 and Ki-67, the cells that expressed CD30 also expressed Ki-67.

**Conclusions:**

The present study identified for the first time the existence of CD30 molecules in the basal epithelium of a pterygium. The fact that the positivity to Ki-67 in the basal epithelium of the pterygium correlated with the CD30 reactivity suggested that this protein could be associated with the uncontrolled cell proliferation of the epithelium in this pathology. The CD30 molecule could therefore be a suitable target to be inhibited using chimerical antibodies in pterygium diseases.

## Introduction

A pterygium is one of the most frequent diseases in ophthalmology. This lesion is characterized by fibrovascular neoformation from the bulbar conjunctiva into the cornea and is composed of epithelium and highly vascular, subepithelial, loose connective tissue. The pterygium has been considered a chronic degenerative condition. Although the pathogenesis of pterygium is not clearly understood, the immunopathological process behind a pterygium has been investigated [1].

Certain findings concerning common features in a pterygium and neoplasia have been proposed, raising the possibility that a pterygium is a neoplastic-like growth disorder [2]. The finding of tumor markers in the biopsies of pterygium lesions such as the p53 tumor suppressor gene, increased cell cycle proteins such as Ki-67 and cyclin D1 proteins, and a decrease of cyclin dependent kinase inhibitor p27 strengthen the hypothesis that a pterygium resembles a tumor-like lesion [3]. Other evidence includes the overexpression  in a pterygium of the cyclooxigenase-2 protein [4], which is associated with UV-induced cutaneous tumorigenesis [5, 6].

The CD30 molecule is a protein that belongs to the tumor necrosis factor receptor family. It was identified for the first time on Reed-Sternberg cells of Hodgkin’s disease [7]. To date, CD30 has been recognized as a pleiotropic molecule that may drive either cell proliferation or apoptosis depending on the microenvironment, and it has clearly been associated with the etiology of diverse neoplastic diseases, including intraocular T-cell lymphoma [8]. Although CD30 protein expression is predominantly in lymphoid cells, such as activated T and B cells, recent findings have determined that the expression of the CD30 molecule has been identified in newborn non-neoplastic skin basal epithelial cells [9], suggesting that CD30 is also expressed by epithelial proliferating and differentiating cells for which the origin is not lymphoid. The association of CD30 expression with the progression of tumors and diseases other than neoplasias has been described as allergic and infectious diseases [10, 11].

On the other hand, Ki-67 is a molecule associated with cell proliferation and the expression of this molecule in conjunction with other molecules, such as COX-2, has recently been related to the development of preneoplasic lesions [12].

In order to establish new knowledge regarding the immunopathology of a pterygium, the aim of this work was to determine the expression and tissue localization of the CD30 molecule in biopsies from a primary pterygium and to compare it to healthy conjunctiva.

## Methods

**Figure 1:**
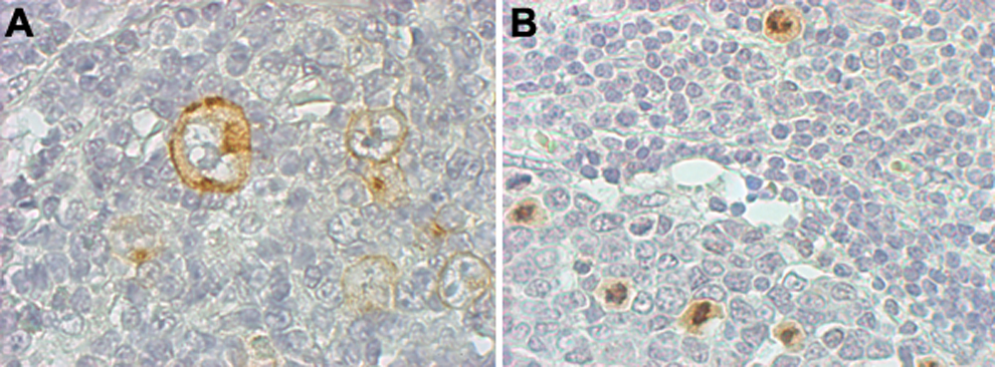
Immunohistochemistry for CD30 and Ki-67. Reed-Sternberg cells are positive for CD30 identified in Hodgkin’s disease (**A**). Nuclear expression of Ki-67 identified in human tonsils (**B**). Both slides were counterstained with Mayer’s hematoxylin (400×).

Twenty-five primary pterygia and 10 healthy conjunctivas were collected after informed consent was obtained from each patient who underwent a surgical procedure. Samples of healthy conjunctiva were obtained from the same patients who underwent a pterygium surgical excision. The biopsies of the healthy conjunctivas were taken from the autografts obtained from the superior bulbar conjunctiva and, unfortunately, 15 out of the 25 specimens were not sufficient for this study. The excision of the pterygium was performed simply, without using mytomicin C or radiation in the surgical procedure. Tissue segments were fixed by 10% formalin overnight and processed for paraffin embedding. Sections of 5 µm were cut and mounted on glass polylysine-charged slides. All slides were then deparaffinized and rehydrated with a gradient of ethanol concentrations. Antigen retrieval was performed by heating the samples in 10 mmol/l citrate buffer (pH 6.0). Samples were washed with 0.1%Tween-PBS (pH 7.3); this buffer was used for all subsequent washes. Primary antibodies were the ready-to-use mouse anti-human CD30 monoclonal antibody (Dako, Glostrup, Denmark) and the mouse anti-human Ki-67 monoclonal antibody (Biocare Medical, Concord, CA) diluted at 1:50; both primary antibodies were incubated at room temperature (RT) for 30 min. Samples were washed twice. All samples were incubated for 30 min with universal biotinylated secondary antibodies at RT. The samples were washed twice and a final incubation of 30 min at RT was performed using streptavidin-peroxidase. Signals were developed using 3, 3’-diaminobenzidine for 5 min and counterstained with Mayer’s hematoxylin (Dako, Glostrup Denmark). Negative controls were performed by leaving out the primary antibody. Protein expression of CD30 was identified in Hodgkin’s lymphoma (Figure 1A) and was used as a positive control for this molecule. Similarly, human tonsils were used as positive controls for Ki-67 immunostaining (Figure 1B). At least 100 pterygium basal epithelial cells in four microscopic fields from the tissue were counted. Positive staining cells were noted by their labeling index as a percentage in each specimen, and the measurements were averaged.

## Results

### Normal bulbar conjunctiva

**Figure 2:**
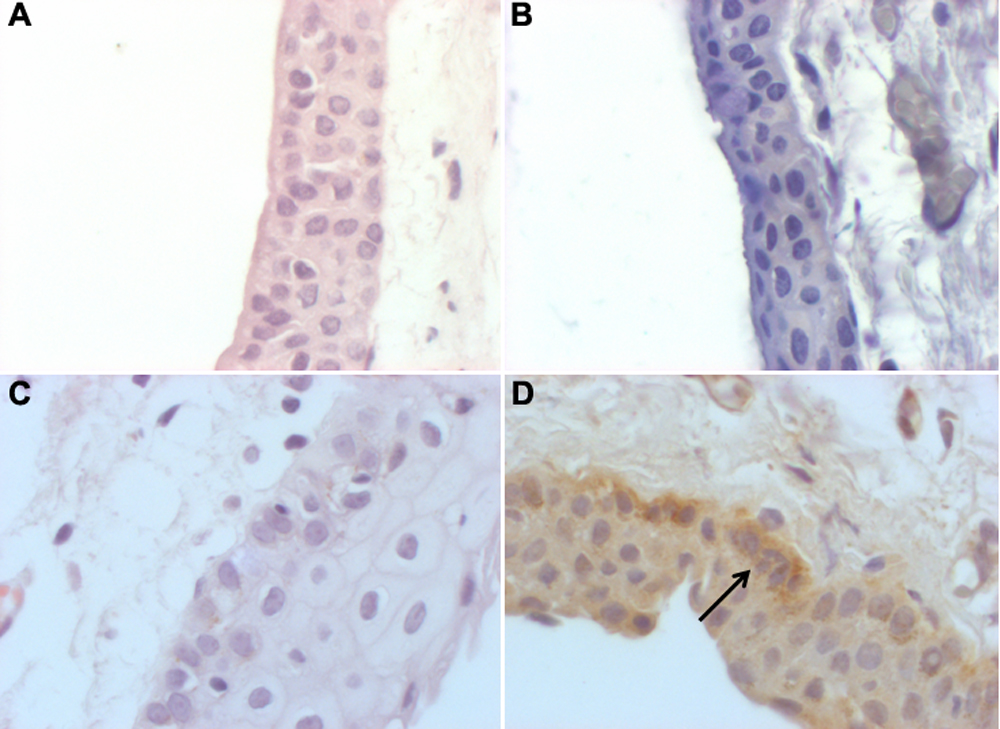
Hematoxylin and eosin staining for healthy conjunctiva (**A**) and pterygium (**C**) and immunoreactivity for CD30 in healthy conjunctiva (**B**) and pterygium (**D**). The arrow indicates the strong immunoreactivity in the basal epithelium (400×).

Conjuctival epithelium consisted of several layers of round cells without keratinization; few goblet cells were found intermingled (Figure 2A,B). Weak immunoreactivity to CD30 was identified in three (30%) normal conjunctivas stained mainly in the basal epithelium and only two (20%) of the three cases of normal bulbar conjunctiva were identified in the stromal side of the tissue. Immunoreactivity to Ki-67 was not found in all of the conjunctivas studied in the epithelial cells or in the stromal side (data not shown).

### Pterygium

**Figure 3:**
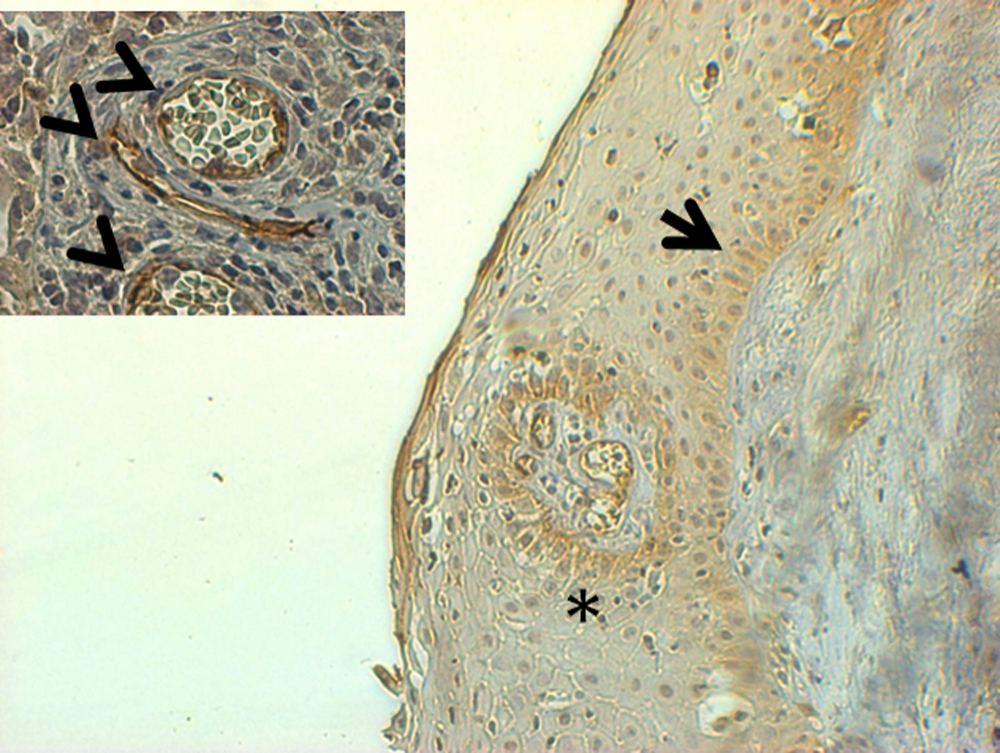
Immunohistochemistry with the CD30 antibody of a pterygium. Representative microphotograph (100×) of a pterygium stained with CD30. The arrow indicates the strong immunoreactivity of CD30 predominantly in basal epithelium in comparison to the weak reactivity to CD30 in parabasal and superficial epithelium. The asterisk indicates perivascular immunostaining with strong immunoreactivity to CD30. The strong perivascular staining of CD30 is shown by arrowheads in the inset at higher magnification (400×).

Histological findings showed that epithelial cells consisted of several layers without nuclear atypia and goblet cell hyperplasia was also observed. In the stroma, numerous microvessels and fibroblasts with elastic degeneration were identified (Figure 2C). Unlike normal bulbar conjunctiva, a strong immunoreactivity to CD30 was identified mainly in the basal epithelium (Figure 2D) and in the parabasal and superficial epithelium; however, the expression was not as strong and was presented in fewer studied pterygia cases. When analysis was performed on the stroma of the pterygium, infiltrating cells were identified. From these cells, some presented positive immunoreactivity to CD30. Likewise, the perivascular immuno-reactivity of CD30 was also observed. The staining pattern observed was granular with cytoplasmic and perinuclear localization (Figure 3 and inset).

**Figure 4:**
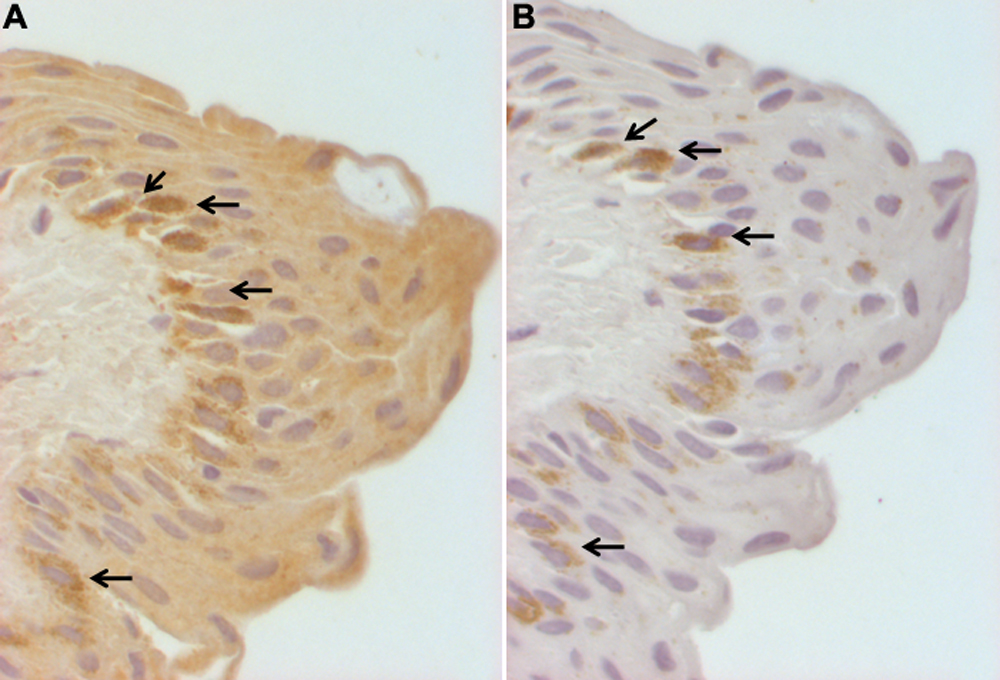
Immunohistochemistry for CD30 and Ki-67 in serial sections of pterygium tissue. Representative microphotograph of CD30 immunopositive cells are shown in **A**, a serial section is shown in **B** for the nuclear localization of Ki-67. Note that the arrows indicate the same cells that are positive to both antigens (400×).

The expression of the Ki-67 antigen was also observed in all pterygia stained with this antibody. Ki-67 staining was confined to the nucleus of the cells from the basal epithelium. No other structures presented immunoreactivity to the Ki-67 antibody. When serial sections were performed, the same cells were stained with both CD30 and Ki-67antibodies identified mostly in the basal epithelium (Figure 4). Table 1 summarizes the number of immunopositive cells.

**Table 1:** Number of cells positive and negative to both CD30 and Ki-67 on basal epithelium of pterygium.

**CD30+**	**CD30-**	**Ki-67+**	**Ki-67**
65% (±3.5%)	38.3% (± 3.1%)	63.3% (±7.3%)	36.6% (±7.3%)

## Discussion

In the present study, CD30 was identified preferentially in pterygium tissue compared with normal conjunctiva, and CD30 expression was demonstrated preferentially in pterygium basal epithelium, stromal infiltrating cells, and perivascular areas. Similarly, Ki-67 was localized in the pterygium epithelial basal cells and the positivity was confined to the nucleus of the cells. This immunostaining was not observed in healthy conjunctivas. There are many studies that tried to identify the etiology of pterygium disease with vast evidence that a pterygium is a growth disorder resulting from uncontrolled cell proliferation rather than a degenerative process [13]. The expression of the CD30 molecule has been widely studied in neoplastic diseases, such as lymphomas [14-16], and it has recently been identified in non-lymphoid tissue as skin associating its expression with proliferation and differentiation [9]. Although the CD30 expression has been identified as transmembranal [17], the staining pattern in a pterygium is mostly cytoplasmic and perinuclear. This result is in accordance with other authors who found that the cells with a positive reaction to CD30 in epithelial cells from skin manifested a fine brown nuclear reaction [9], suggesting a different localization of this antigen rather than a membrane expression, which is supported by other authors who have shown a cytoplasmic and Golgi localization of the CD30 molecule [18, 19].

Pterygium tissue consists of cell hyperplasia, including goblet cell hyperplasia [20]. The goblet cells of a pterygium and healthy conjunctiva were negative to both CD30 and Ki-67 immunostaining, suggesting that the proliferation action of CD30 is not driven directly by CD30’s function. In this study, Ki-67 was expressed in basal epithelium; these results are in accordance with those found by Kase et al., who demonstrated that the expression of Ki-67 in a pterygium was confined to epithelial cells rather than fibroblasts and goblet cells [3]. When the immunohistochemistry of both CD30 and Ki-67 on pterygium samples was performed, the same cells were stained with these two antibodies, suggesting a strong correlation between CD30 and cell proliferation.

Unlike in goblet cells, the strong immunoreactivity of CD30 molecule was observed in the basal epithelium of  a pterygium, suggesting that these cells were proliferating and probably the abnormal expression of CD30 in these cells determined the uncontrolled cell proliferation reported in a pterygium [13]. As supported by other authors, CD30 has been identified in epithelial cells in the basal germinative layer of the epidermis [9] and placenta [21-23], which suggested that this molecule was expressed by epithelial proliferating and differentiating cells of other than lymphoid origin.

In this study, CD30 was identified in some of infiltrating cells. Whether these cells possess proliferating activity is still being studied. Detection of CD30 on perivascular regions could be associated with infiltrating lymphocytes that present this antigen [24]; however, the possibility of immunoreaction directly on the endothelial cells cannot be ruled out [25]. The evidence that CD30 protein expression in epithelial cells from a pterygium was associated with proliferation rather than apoptosis suggested that this molecule was a potential target for biological therapy of treating a pterygium with chimerical anti-CD30 monoclonal antibodies [26] and this could lead to new perspectives in the therapy of this common disease.
